# Replication and expansion of epigenome-wide association literature in a black South African population

**DOI:** 10.1186/s13148-019-0805-z

**Published:** 2020-01-07

**Authors:** H. Toinét Cronjé, Hannah R. Elliott, Cornelie Nienaber-Rousseau, Marlien Pieters

**Affiliations:** 1 Centre of Excellence for Nutrition at the North-West University Potchefstroom Campus, Potchefstroom, 2520 South Africa; 2 MRC Integrative Epidemiology Unit, University of Bristol, Bristol, BS8 2BN UK; 3 Population Health Sciences, Bristol Medical School, University of Bristol, Bristol, BS8 2BN UK

**Keywords:** Ancestry, DNAm, EPIC, Epigenetic epidemiology, EWAS, Methylation, NCD, PURE

## Abstract

**Background:**

DNA methylation is associated with non-communicable diseases (NCDs) and related traits. Methylation data on continental African ancestries are currently scarce, even though there are known genetic and epigenetic differences between ancestral groups and a high burden of NCDs in Africans. Furthermore, the degree to which current literature can be extrapolated to the understudied African populations, who have limited resources to conduct independent large-scale analysis, is not yet known. To this end, this study examines the reproducibility of previously published epigenome-wide association studies of DNA methylation conducted in different ethinicities, on factors related to NCDs, by replicating findings in 120 South African Batswana men aged 45 to 88 years. In addition, novel associations between methylation and NCD-related factors are investigated using the Illumina EPIC BeadChip.

**Results:**

Up to 86% of previously identified epigenome-wide associations with NCD-related traits (alcohol consumption, smoking, body mass index, waist circumference, C-reactive protein, blood lipids and age) overlapped with those observed here and a further 13% were directionally consistent. Only 1% of the replicated associations presented with effects opposite to findings in other ancestral groups. The majority of these inconcistencies were associated with population-specific genomic variance. In addition, we identified eight new 450K array CpG associations not previously reported in other ancestries, and 11 novel EPIC CpG associations with alcohol consumption.

**Conclusions:**

The successful replication of existing EWAS findings in this African population demonstrates that blood-based 450K EWAS findings from commonly investigated ancestries can largely be extrapolated to ethnicities for which epigenetic data are not yet available. Possible population-specific differences in 14% of the tested associations do, however, motivate the need to include a diversity of ethnic groups in future epigenetic research. The novel associations found with the enhanced coverage of the Illumina EPIC array support its usefulness to expand epigenetic literature.

## Background

The role of epigenetics in the aetiology of non-communicable diseases (NCDs) is of interest owing to its valuable addition to the limited variance of disease risk explained by genetics alone [1]. The modifiable nature of the epigenome also offers opportunities to predict, detect and prevent lifestyle-related diseases [2]. DNA methylation (DNAm) is the most intensively researched epigenetic modification, partly because of its ease of measurement from stored samples commonly collected in epidemiological studies. A number of robust associations between differentially methylated cytosine-guanine dinucleotides (CpGs) and NCD-related traits or exposures have been reported [3–7]. Epigenetic research has allowed for richer insight into the origin and progression of complex diseases, and is expected to continue doing so, thereby enhancing our ability to combat the continued rise in NCD prevalence [2, 8].

Despite its importance in the global context of NCDs, current epigenetic literature remains limited by the lack of ethnic diversity, with most investigating associations between DNAm and health outcomes/traits within European (EU) populations. Although several large-scale epigenome-wide association studies (EWASs) have used data collected from African American (AA) individuals [4, 5, 9], information on continental African populations remain particularly limited. Sub-Saharan Africans are known to be genetically different from AA individuals, who typically stem from West African ancestors, with varying levels of admixture [10]. Because DNAm differences have been reported among ethnic groups [11–13], the degree to which current EWAS results can be extrapolated to other populations, including Sub-Saharan Africans, remains to be established. Understanding the degree of generalisability of EWAS results to different ethnicities informs one whether existing knowledge can be extrapolated to understudied ethnic groups or whether additional research is needed in these populations, where resources are often limited [14].

To this end, we replicated data extracted from the EWAS Catalogue (http://www.ewascatalog.org) on traits related to NCDs (alcohol consumption, smoking status, body mass index (BMI), waist circumference (WC), high-density lipoprotein cholesterol (HDL-C), low-density lipoprotein cholesterol (LDL-C), total cholesterol (TC), triglycerides (TG), C-reactive protein (CRP) and age), in a subset of Batswana men from the North-West (NW) province of South Africa, who participated in the international Prospective Urban and Rural Epidemiology study (PURE-SA-NW). In doing so, we evaluated the reproducibility of previous EWAS findings in a Sub-Saharan African population that has never been investigated before. In addition, because the majority of EWASs to date have been conducted using the older Illumina 450 K BeadChip, our secondary aim was to report novel DNAm associations by using the new Illumina MethylationEPIC platform, to extend existing knowledge on methylation and traits related to NCDs in this population [15].

## Results

For each trait, we report the degree of replication between EWAS findings in the PURE-SA-NW cohort and the reference studies identified, using the EWAS catalogue (complete test statistics in Additional file 1). In cases where the reference study included cohorts of different ancestries, the PURE-SA-NW cohort was compared to these ancestries separately. We first report the agreement between the effect sizes obtained in the PURE-SA-NW data and the reference studies for all the tested CpGs per trait, to evaluate the overall consensus between the studies (PURE-SA-NW vs. reference study). We then examine the similarity between studies at the individual CpG level by determining whether or not the individual PURE-SA-NW association’s confidence intervals (CIs) overlap with those of the reference study. This allows us to identify systematic differences (e.g. attributable to exposure variation) between cohorts before investigating differences at an individual CpG level (e.g. attributable to site-specific genetic variation). To permit further investigation of individual CpG association differences, we inspect probes previously identified to measure methylation at polymorphic sites of which either the global minor allele frequency (MAF) is higher than 1% [16], or variation has been documented in Africans, specifically [17] (Additional file 1). Probes identified to hybridise to multiple genomic regions or to be cross-reactive are also noted [18]. Replication analyses are followed by a report of any methylation associations of newly investigated EPIC probes and novel 450K associations (of 450K probes present on the EPIC array used here), where applicable (Additional file 2). Table 1 provides the descriptive statistics for (i) the PURE-SA-NW cohort for traits used as covariates in the models, (ii) the trait of interest as reported by the EWAS catalogue reference study and (iii) the PURE-SA-NW cohort trait of interest reported in the same unit as in the specific reference study. For the different traits, the sample size here differs because we applied the specific inclusion criteria of the respective reference studies to our population to permit comparison (Additional file 1).

**Table 1:** Descriptive characteristics of the study and reference cohorts

Trait	PURE-SA-NW	Reference study	Comparative PURE-SA-NW	Reference study citation
*N*	120	See Additional file 1		
Age (years)	64 [55–70]	62 [58 − 67]	64 [55–70]	[19]
BMI (kg/m^2^)	22.5 ± 4.9	27.6 ± 4.4^a,e^	22.4 ± 5.0	[20]
		27.7 ± 4.5^b,e^		
WC (cm)	83.8 ± 12.8	101 ± 15.1^c,e^	83.6 ± 12.7	[9]
		97 ± 16^d,e^		[21]
Physical activity (index)	2.41 ± 0.94			
Smoking status [*N* (%)]				
Never smoker	56 (47)	6956 (74)^b,d,e^	56 (48)	[4]
Current smoker	61 (51)	2433 (26)^b,d,e^	61 (52)	
Ever smoker	64 (53)			
Alcohol use [*N* (%)]				
Never user	56 (47)			
Ever user	64 (53)			
Alcohol consumption (g/day)	16.7 ± 36.6	1.3 (0, 301)^c^	0 (0, 240)	[5]
		5.6 (0, 181)^d^		
CRP (mg/L)	9.7 ± 27.2	6.2 ± 8.8^c,e^	9.9 ± 27.5	[22]
		3.3 ± 5.6^d,e^		
TC (mg/dL)	171 ± 41.6	207 ± 37.1^d,e^	171 ± 41.6	[3]
LDL-C (mg/dL)	96.5 ± 35.7	125 ± 30.9^d,e^	96.5 ± 35.7	
HLD-C (mg/dL)	54.1 ± 22.7	57.0 ± 16.8^d^	54.1 ± 22.7	
TG (mg/dL)	48.5 ± 30.5	126 ± 69.0^d,e^	48.5 ± 30.5	
Education [*N* (%)]				
None	26 (22)			
1–7 years of schooling	66 (55)			
8–12 years of schooling	28 (23)			
Blood cell type proportions (%)				
B cells	0.04 ± 0.02			
CD4 T cells	0.11 ± 0.04			
CD8 T cells	0.11 ± 0.06			
Granulocytes	0.47 ± 0.11			
Monocytes	0.09 ± 0.02			
Natural killer cells	0.11 ± 0.03			

*BMI* body mass index, *CRP* C-reactive protein, *HDL*-*C* high-density lipoprotein cholesterol, *LDL*-*C* low-density lipoprotein cholesterol, *TC* total cholesterol, *TG* triglycerides, *WC* waist circumference. Values are presented as median [IQR], mean ± standard deviation, *N* (%) or median (minimum, maximum). Blood cell proportions were determined using methylation-based estimates [23]

^a^Indian Asian ancestry

^b^European American ancestry

^c^African American ancestry

^d^European ancestry

^e^Population means differ between the reference study and comparative PURE-SA-NW study population at *p* < 0.05 following Bonferroni adjustment

Comparatively, our study population had a more favourable body composition and blood lipid profile, but a much higher CRP concentration than those included in the reference studies [3, 9, 22]. The proportion of current smokers in our study population was twice as high as the reference cohort [4], and they consumed larger volumes of alcohol than the EU, but less than the AA reference cohorts [5]. The remaining traits were similar between our population and that of the reference studies.

### Alcohol consumption

**Fig. 1:**
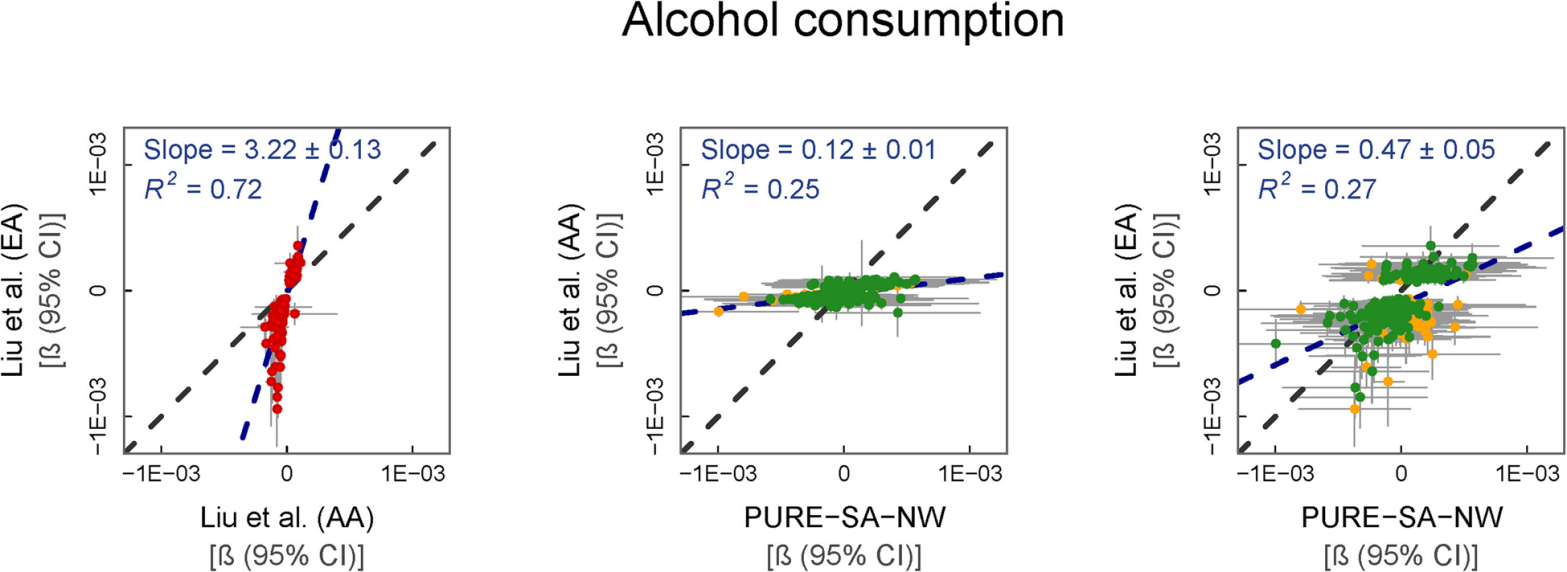
% Methylation change per gram of alcohol intake. From left to right: (i) reference AA vs. EA data (247 CpGs), (ii) PURE-SA-NW vs. AA data (228 CpGs) and (iii) PURE-SA-NW vs. EA data (228 CpGs). Model used: methylation ~ alcohol consumption + age + BMI + cell counts + surrogate variables. Reference data: Liu et al. (2018). Green data points represent CpGs where the 95% CIs for effect size estimates in each sample group overlap. Yellow data points represent CpGs where the 95% CIs for effect size estimates in each sample group do not overlap. Red data points represent the comparison of effect sizes within the reference cohorts. Black dashed line: line of equality. Blue dashed line: regression line

Ancestry-stratified (European American (EA) and AA) findings from the meta-analysis by Liu et al. [5] on the association of alcohol consumption (g/day) with differential methylation at individual CpGs were compared with those from the PURE-SA-NW (Fig. 1). In the study of Liu et al. [5], alcohol consumption was more strongly associated with DNAm in AA than EA individuals (regression slope = 3.2, *p* = 8.6 × 10^−70^). Effect sizes in the PURE-SA-NW cohort were larger than in either of the reference groups (regression slope = 0.12, *p* = 3.2 × 10^−16^ and 0.47, *p* = 3.2 × 10^−17^ for the AA and EA comparisons, respectively).

Individual association results showed stronger similarity between the PURE-SA-NW and the AA than with the EA findings. Overall, 361 CpGs were investigated (two unique AA, 131 unique EA and 228 associations reported for both ethnicities). Out of the 230 association tests to compare the AA reference cohort to the PURE-SA-NW data, 93% (213) of the regression CIs overlapped, compared to 80% (287) of the 359 comparisons between the EA and PURE-SA-NW. Where CIs did not overlap, directional consistency was nevertheless observed with the exception of the associations for cg15636519 (EA and AA comparisons), cg08471846 (EA comparison only) and cg21227253 (EA comparison only) with alcohol consumption (Additional file 1a). Data from the Biobank-based Integrative Omics Studies (BIOS) Consortium indicated that, apart from cg08471846, methylation quantitative trait loci (mQTLs) have been identified for each of these CpGs with absolute reported Z-scores ranging from 4.15 to 12.9 [24, 25]. Data from the 1000 Genomes project support that the differences observed here could be partly influenced by ancestry-specific genetic variance; for example, the MAF of rs7153432 (*cis* mQTL for cg21227253) is 18% in Africans and 40% in Europeans [26].

The EWAS conducted on alcohol consumption in the PURE-SA-NW cohort resulted in 19 genome-wide significant findings (*p* < 9.4 × 10^−8^), 11 of which were newly investigated EPIC probes and eight were part of those previously investigated by 450K probes, that were present on the EPIC array, but failed to reach association thresholds in other cohorts (Additional file 2a). Table 2 provides the test statistics for these CpGs.

**Table 2:** EWAS CpG-alcohol consumption associations *p* < 9.4 × 10^−8^

ProbeID	Location	Gene	Region	β	SE	*p*	% Variance explained	*Χ*^*2*^ *p* value
cg13153796^a^	14:101405628	*SNORD*113-6	TSS1500	− 6.78E–04	8.45E–05	2.2E–11	29.4 (38.4)	3.8E–12
cg00712390^a^	17:79373624	*BAHCC*1	1stExon	8.08E–04	1.14E–04	9.7E–10	37.5 (47.0)	5.6E–18
cg05706661	7:36134301	*LOC*101928618	TSS1500	− 1.05E–03	1.51E–04	2.0E–09	17.6 (57.1)	6.7E–12
cg24252287^a^	17:40250379			1.48E–04	2.21E–05	4.8E–09	36.5 (41.8)	7.7E–15
cg12177743^a^	11:113185079	*TTC*12	TSS200	1.59E–04	2.41E–05	7.5E–09	13.4 (23.4)	2.8E–05
cg19323439	17:9136232	*NTN*1	Body	5.06E–04	7.93E–05	1.9E–08	14.1 (59.0)	2.1E–08
cg19683675^a^	5:142077712	*FGF*1	TSS200	− 1.13E–03	1.78E–04	2.0E–08	35.1 (43.6)	2.7E–15
cg08333974	12:1956337	*CACNA*2*D*4	Body	− 1.24E–03	1.95E–04	2.2E–08	25.8 (38.3)	8.3E–12
cg12325997	15:59280148	*RNF*111	1stExon	9.84E–05	1.57E–05	3.2E–08	10.5 (58.4)	9.4E–08
cg19642811	13:95453039	*LOC*101927284	Body	− 6.02E–04	9.64E–05	3.4E–08	19.3 (37.3)	2.3E–08
cg06943216	8:102683096			− 1.33E–03	2.13E–04	3.5E–08	17.5 (33.9)	4.3E–08
cg26187237^a^	2:217498574	*IGFBP*2	1stExon	4.19E–04	6.72E–05	3.6E–08	15.5 (53.0)	2.2E–09
cg16358446^a^	1:1534984			8.10E–05	1.31E–05	4.4E–08	43.8 (52.4)	1.9E–21
cg08724692	6:133646558	*EYA*4	Body	− 6.26E–04	1.03E–04	6.4E–08	10.3 (43.4)	1.2E–06
cg08035774	9:136600662	*SARDH*	5′UTR	− 1.12E–03	1.85E–04	7.5E–08	23.6 (32.8)	6.3E–10
cg18780412^a^	3:179755086	*PEX*5*L*	TSS1500	6.36E–04	1.06E–04	8.6E–08	27.0 (33.5)	6.4E–11
cg15942324	1:38482118	*UTP*11*L*	Body	− 6.63E–04	1.10E–04	8.8E–08	23.3 (33.2)	3.4E–09
cg25278025	2:103378026	*TMEM*182	TSS1500	5.99E–04	9.98E–05	8.8E–08	15.4 (26.0)	5.0E–06
cg22572934^b^	5:173171061	*LINC*01484	Body	− 1.21E–03	2.02E–04	9.3E–08	13.3 (24.3)	5.5E–05

Model: methylation ~ alcohol consumption (g/d) + age + BMI + smoking + cell counts + surrogate variables

^a^450K probes

^b^Probe that should be interpreted with caution owing to the presence of genomic variance at probe measurement site [17]

The percentage variance explained reflects the added value of alcohol consumption to the variance in CpG methylation, reported as percentage explained by alcohol as an added exposure (percentage variance explained by the total model). *Χ*^*2*^ *p* value = Chi-square *p* value when the regression models with and without alcohol consumption are compared

The proportion of methylation variance of these CpGs explained by including alcohol consumption in the model methylation ~ age + BMI + cell counts + smoking status, ranged from 10.3 to 43.8%. When alcohol consumption was used as the outcome variable, the addition of these 19 probes to the regression model, increased the percentage of alcohol consumption variance explained by 57% (adjusted *R*^2^ = 0.05 before and 0.62 after including the CpGs, *p* = 5.5 × 10^−26^).

### Smoking status

The association of smoking status with the DNAm of 3618 CpGs in the PURE-SA-NW cohort was compared to a multi-ethnic (EA and AA) EWAS conducted by Joehanes et al. [4]. ‘Current’ users in the PURE-SA-NW cohort included individuals regularly smoking any bought or self-made tobacco product (commercial cigarettes, bidis, pipes and cigars). Joehanes et al. [4], however, restricted the definition of ‘current’ smokers to those specifically reporting cigarette use. Regardless of the discrepancy in the product smoked, results from the respective EWASs were fairly similar. No ancestral comparisons were made by Joehanes et al. [4], who combined data from a number of different ethnic groups in a meta-analysis.

**Fig. 2:**
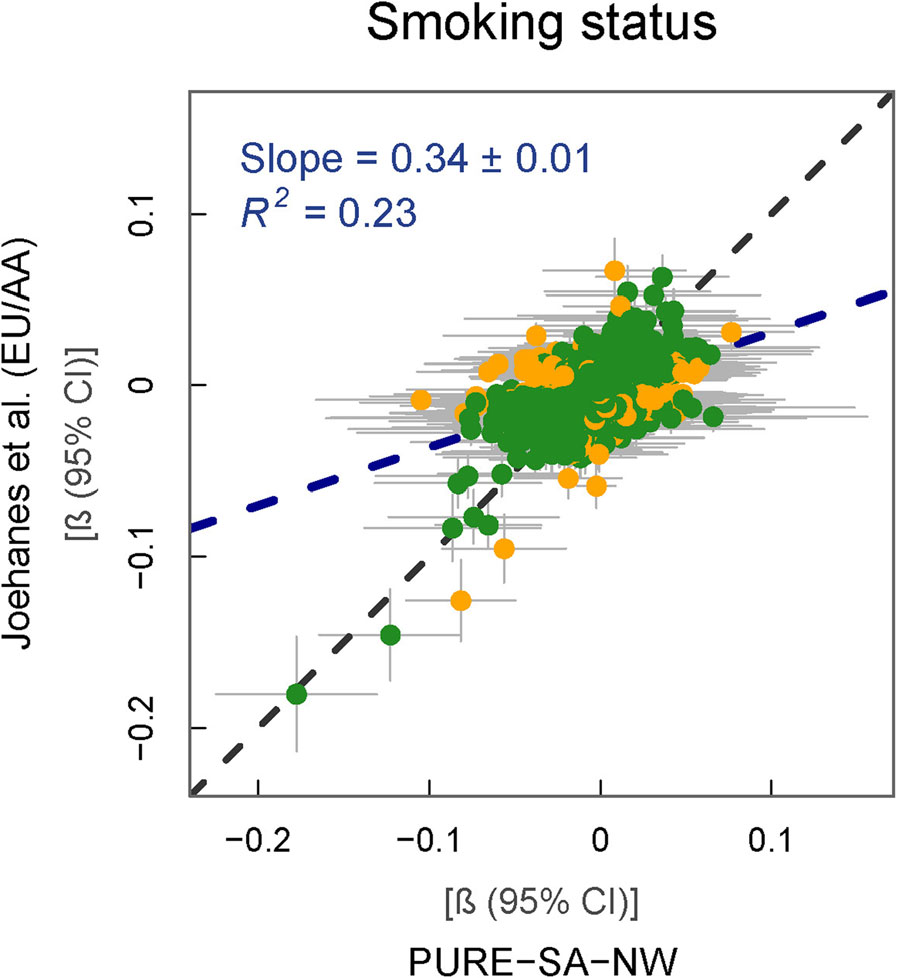
% Methylation difference between current and never smokers in reference vs. PURE-SA-NW data. Model used for PURE-SA-NW EWAS: methylation ~ smoking + age + cell counts + surrogate variables. Green data points represent CpGs where the 95% CIs for effect size estimates in each sample group overlap. Yellow data points represent CpGs where the 95% CIs for effect size estimates in each sample group do not overlap. Black dashed line: line of equality. Blue dashed line: regression line

Effect sizes were generally larger in the PURE-SA-NW than in the reference data (regression slope = 0.34, *p* = 1.7 × 10^−206^). Of the 3618 CpGs tested for their independent association with smoking status, 3315 (92%) of the regression β 95% CIs overlapped and 269 were directionally consistent between cohorts (Fig. 2). Only 34 CpGs showed a difference in the direction of effect between the findings of Joehanes et al. [4] and the PURE-SA-NW cohort (Additional file 1b). Thirteen of these probes measure methylation at polymorphic sites, 20 have *cis-*mQTLs and five have *trans-*mQTLs, all of which with differing AA and EU ancestry MAFs, suggesting that genetic variation between cohorts could drive some of the dissimilarities observed [24–26]. No novel associations with smoking were identified and the only genome-wide significant CpG association was for a previously identified CpG (cg05575921) that was associated with a 17% (*p* = 4.2 × 10^−10^) reduction in DNAm in *current* smokers compared to participants who had *never* smoked (Additional file 2b).

### Body mass index

**Fig. 3:**
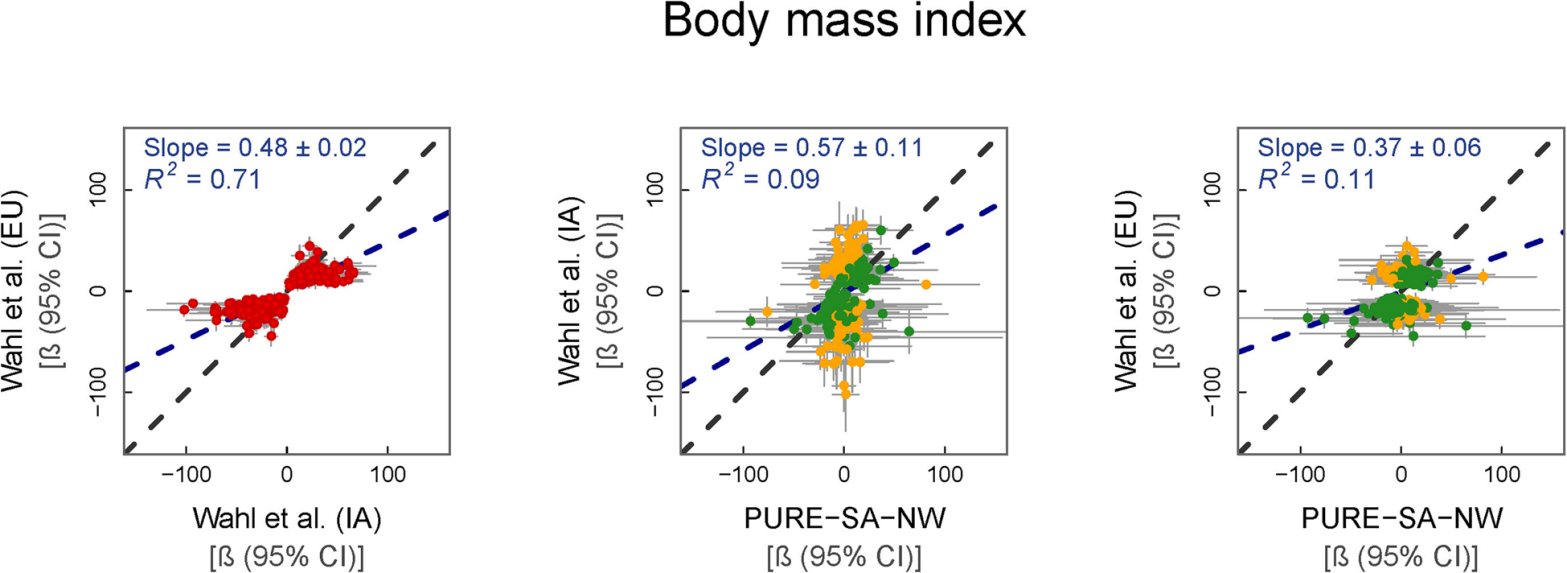
Change in BMI (kg/m^2^) per % methylation change. From left to right: (i) reference EU vs IA data, (ii) IA vs. PURE-SA-NW data and (iii) EU vs. PURE-SA-NW data. Model used for PURE-SA-NW EWAS: BMI ~ methylation + age + smoking status + alcohol consumption + physical activity + cell counts + surrogate variables. Reference data: Wahl et al. (2017). Green data points represent CpGs where the 95% CIs for effect size estimates in each sample group overlap. Yellow data points represent CpGs where the 95% CIs for effect size estimates in each sample group do not overlap. Red data points represent the comparison of effect sizes within the reference cohorts. Black dashed line: line of equality. Blue dashed line: regression line

We replicated findings from the largest EWAS on BMI conducted to date, that of Wahl et al. [20]. These authors investigated the relationship of methylation with BMI in individuals of Indian Asian (IA) and EU descent. Wahl et al. [20] observed larger effect sizes among the IA than the EU group (regression slope = 0.48, *p* = 4.9 × 10^−72^). PURE-SA-NW data reflected the IA better than the EU data, but in both instances, PURE-SA-NW data showed larger effect sizes than either reference group (regression slope = 0.57, *p* = 6.0 × 10^−7^ and 0.37, *p* = 1.8 × 10^−8^ for IA and EU groups, respectively). However, when comparing the overlap between individual effect estimates, PURE-SA-NW mirrored findings from the EU group better. The 95% CIs of the 265 regression estimates between the cohorts overlapped 55% (147) and 77% (203) of the time when compared with IA data and EU data, respectively (Fig. 3). All regression CIs that did not overlap were directionally consistent between the PURE-SA-NW and reference cohorts. No genome-wide significant associations with BMI were identified (Additional file 2c).

### Waist circumference

**Fig. 4:**
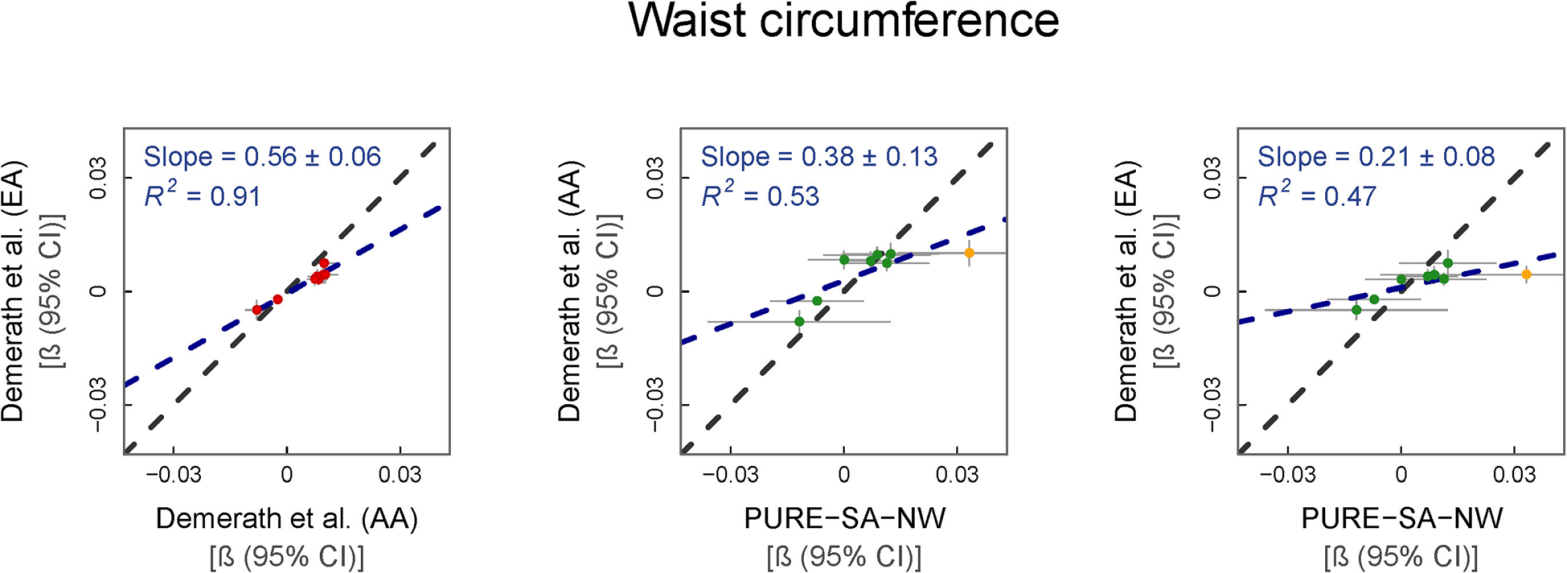
% Methylation change per centimetre change in WC. From left to right: (i) reference EA vs. AA data, (ii) AA vs. PURE-SA-NW data and (iii) EA vs. PURE-SA-NW data. WC was normalised to have a mean of 0 and a standard deviation of 1. Model used for PURE-SA-NW EWAS: methylation ~ WC + age + alcohol consumption + smoking + physical activity + education + cell counts + surrogate variables. Reference data: Demerath et al. (2015). Green data points represent CpGs where the 95% CIs for effect size estimates in each sample group overlap. Yellow data points represent CpGs where the 95% CIs for effect size estimates in each sample group do not overlap. Red data points represent the comparison of effect sizes within the reference cohorts. Black dashed line: line of equality. Blue dashed line: regression line

Eight previously reported associations of WC with DNAm in cohorts of AA and EA descent [9] were replicated in the PURE-SA-NW cohort (Fig. 4). The regression model used to quantify the relationship between WC and DNAm differed between the reference cohort subgroups. In addition to the covariates adjusted for in the EA regression model (age, smoking and white blood cell counts), the AA model also included alcohol consumption status, physical activity, education and household income. The use of the two different models was justified, as it resulted in highly comparable findings between the reference study’s AA and EA groups (*r* = 0.96), with a slightly larger average effect size observed in the EA than in the AA data (regression slope = 0.56, *p* = 0.0001). Applying the fully adjusted (AA) model to the PURE-SA-NW data resulted in a 10.4% increase in average effect size compared to the model used for the EA group, justifying the use of the fully adjusted model in our cohort.

As for the previous traits, larger effect sizes were observed in the PURE-SA-NW than both the AA (regression slope = 0.38, *p* = 0.03) and EA (regression slope = 0.21, *p* = 0.04) cohorts with a closer resemblance to the AA than the EA data (*R*^2^ = 0.53. vs. 0.47). When comparing individual effect estimates between the PURE-SA-NW and reference data, the 95% CIs overlapped in seven of the eight assessed associations in both groups (Additional file 1d). The non-overlapping associations were directionally consistent between studies, overall indicating strong comparability between WC’s association with DNAm across the investigated ancestral groups. The single non-overlapping locus was the same in both ethnic groups compared. This site, cg26403843, is associated with five *cis*-mQTLs and one *trans*-mQTL with absolute Z-scores ranging from 4.9 to 39.8. Population differences between the mQTL-associated SNPs were observed; rs6556405, for example, has a MAF of 26% in Europenas compared to a frequency of 66% in Africans [24–26].

### Blood lipids

**Fig. 5:**
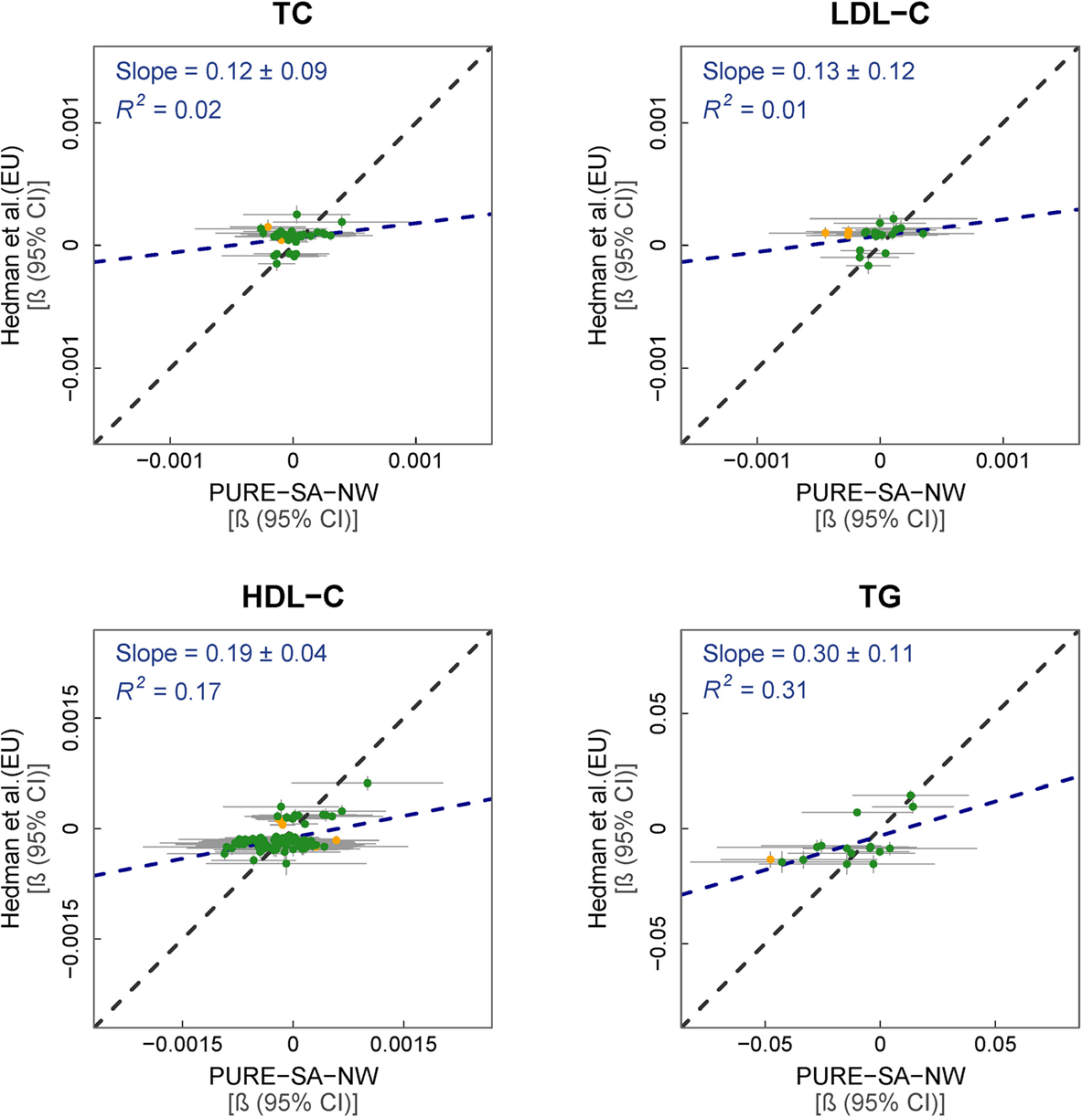
% Methylation change per mg/dL change in lipid concentration in reference vs. PURE-SA-NW data. Models used: methylation ~ lipid (TC, LDL-C, HDL-C or TG) + age + cells + surrogate variables. Green data points represent CpGs where the 95% CIs for effect size estimates in each sample group overlap. Yellow data points represent CpGs where the 95% CIs for effect size estimates in each sample group do not overlap. Red data points represent the comparison of effect sizes within the reference cohorts. Black dashed line: line of equality. Blue dashed line: regression line

Findings from the largest TC, LDL-C, HDL-C and TG EWASs to date, reported by Hedman et al. [3], were compared to those of the PURE-SA-NW cohort. For each of the four lipids, larger effect sizes were observed in the PURE-SA-NW than in the EU reference cohort. The regression slopes when modelling the PURE-SA-NW effect sizes against those of the reference cohorts’ were 0.12 (*p* = 0.18), 0.13 (*p* = 0.27), 0.19 (*p* = 9.9 × 10^−06^) and 0.30 (*p* = 0.01) for TC, LDL-C, HDL-C and TG, respectively (Fig. 5). Effect estimates and 95% CIs overlapped for 38/40 (95%) for TC, 18/21 (86%) for LDL-C, 96/102 (94%) for HDL-C and 15/16 (94%) for TG, of the associations tested (Additional file 1e). Ten of the 12 non-overlapping associations were directionally consistent, leaving only two associations divergent in the direction of effect: cg24939194-HDL-C and cg15878619-TC. Two mQTLs have been identified for cg24939194 (rs748097 and rs2969017), the strongest of which has a MAF of 6% in Africans and 37% in Europeans, indicating that genetic ancestry may be important for the association of cg24939194 with HDL-C [26].

Despite the consistency in the effect sizes between the PURE-SA-NW and the reference data, the large CIs observed in our data do not allow for further interpretation of these findings. There was one genome-wide significant lipid-DNAm association in our cohort (Additional file 2e). High-density lipoprotein cholesterol associated with cg23636606 at a regression β of 2.6 × 10^−04^ ± 4.4 × 10^−05^ (*p* = 4.8 × 10^−08^).

### CRP

**Fig. 6:**
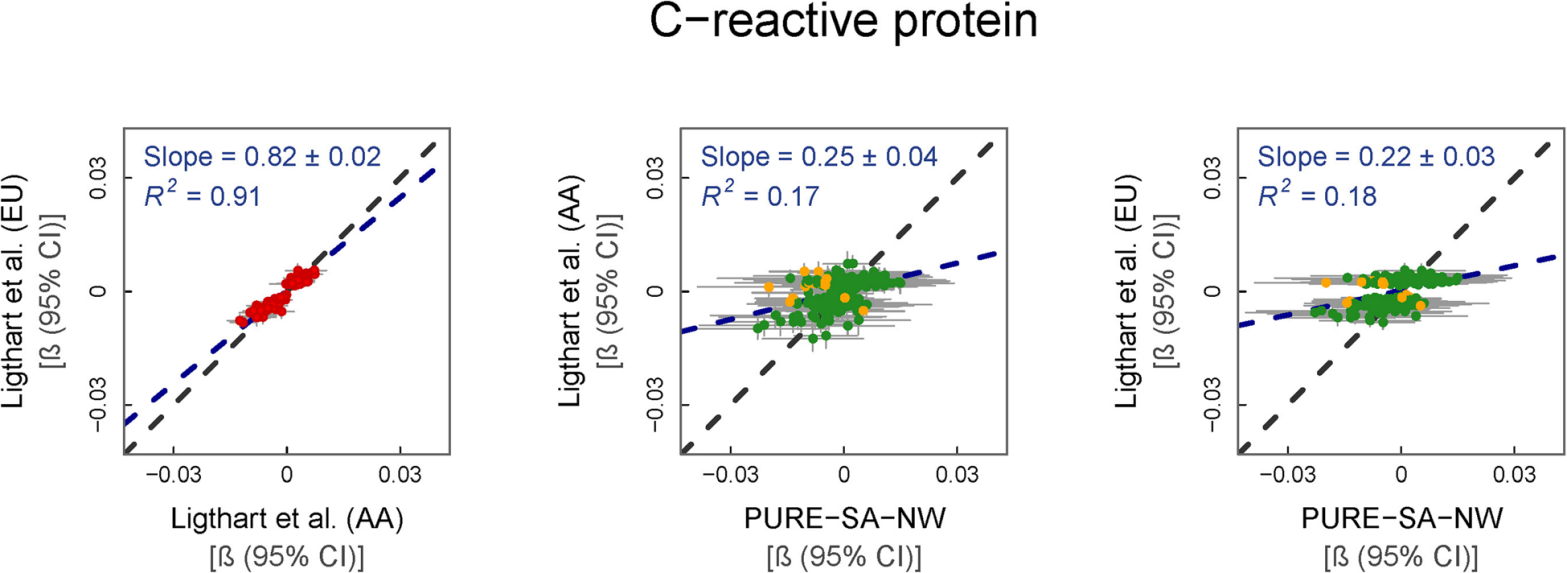
Change in logarithmic CRP (mg/L) per % methylation change. From left to right: (i) reference EU vs. AA data, (ii) AA vs. PURE-SA-NW data and (iii) EU vs. PURE-SA-NW data. Model used for PURE-SA-NW EWAS: methylation ~ CRP + age + smoking + BMI + cells + surrogate variables. Reference data: Ligthart et al. (2016). Green data points represent CpGs where the 95% CIs for effect size estimates in each sample group overlap. Yellow data points represent CpGs where the 95% CIs for effect size estimates in each sample group do not overlap. Red data points represent the comparison of effect sizes within the reference cohorts. Black dashed line: line of equality. Blue dashed line: regression line

Ancestry-stratified (AA and EU) data on the effect of CRP on the DNAm of 207 loci, by Ligthart et al. [22] were compared to PURE-SA-NW. The reference study reported highly comparable effect sizes between the AA and EU ancestral groups (regression slope = 0.82, *p* = 1.25 × 10^−107^), with slightly larger effects observed in the AA group. The comparison of the regression slope of effect sizes between the reference data and our own showed moderately larger effect sizes in the PURE-SA-NW findings than in the reference data, more so for the EU (regression slope = 0.25, *p* = 2.5 × 10^−10^) than the AA (regression slope = 0.22, *p* = 1.3 × 10^−10^) comparison (Additional file 1f). Confidence intervals of the individual effect estimates between the reference and PURE-SA-NW data overlapped for 192 out of the 207 tests (93%) in each ethnicity (Fig. 6). Twenty-two of the 30 non-overlapping associations were directionally consistent. Two CpGs had associations in opposing directions of effects compared to EU (cg01588592 and cg23740758) and three compared to the EU and AA (cg24174557, cg26846781, cg27184903) reference datasets. All the non-overlapping CpGs have *cis-*mQTLs with absolute reported Z-scores ranging from 4.06 to 22.95 [24, 25]. Data from the 1000 Genomes project support the notion that the differences observed here could be partly influenced by ancestry-specific genetic variance: for example, MAF of rs9791189 (*cis-*mQTL for cg23740758) is 12% in Africans and 23% in Europeans [26]. There were no genome-wide significant or novel CRP-DNAm associations in our cohort (Additional file 2f).

### Age

**Fig. 7:**
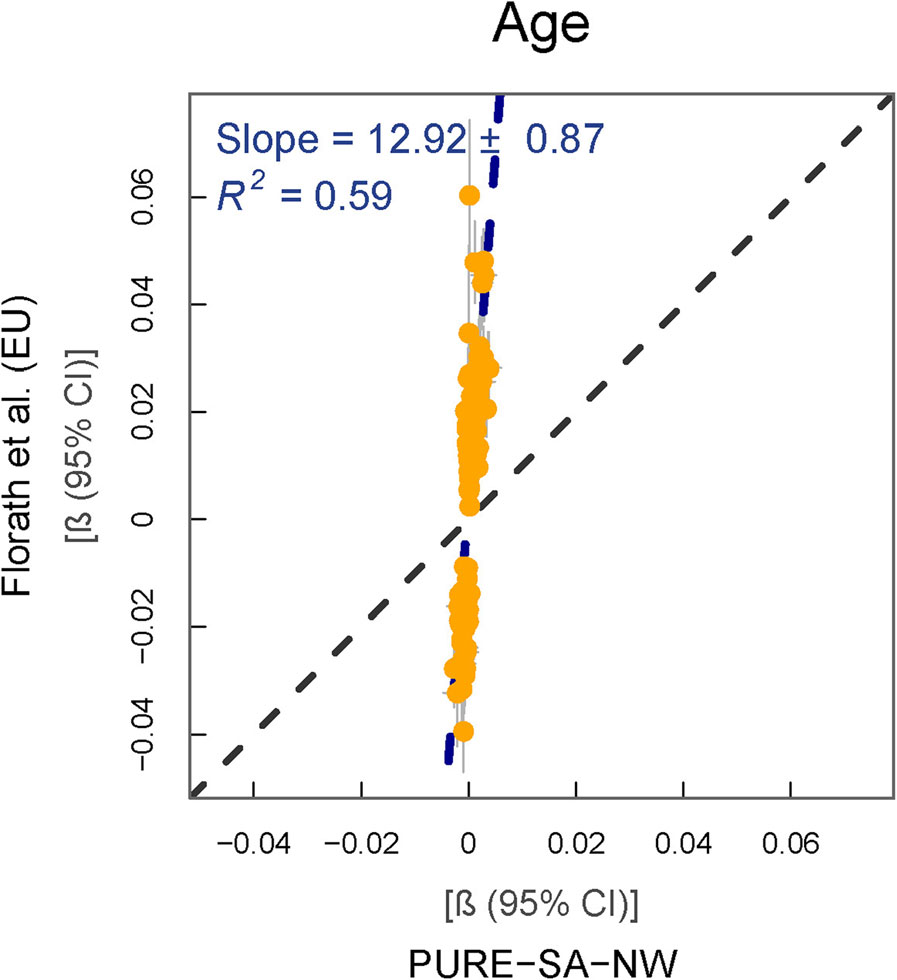
% Methylation change per year of age in reference vs. PURE-SA-NW data. Model used for PURE-SA-NW EWAS: methylation ~ age + smoking + cell counts + surrogate variables. Yellow data points represent CpGs where the 95% CIs for effect size estimates in each sample group do not overlap. Black dashed line: line of equality. Blue dashed line: regression line

Previous findings from EU-based research on the association of age with DNAm of 152 CpGs [19] were compared to those from the PURE-SA-NW cohort (Fig. 7). In contrast to all other traits, a much weaker association between age and DNAm was observed in our data than in the reference data (regression slope = 12.9, *p =* 4.2 × 10^−31^). Although the direction of effects was consistently similar between the two studies, none of the regression CIs overlapped when comparing the individual associations (Additional file 1g).

Formal data on disease diagnosis were not available for the PURE-SA-NW cohort and were, therefore, not included in regression models, as done by Florath et al. [19]. Furthermore, cell counts were not adjusted for in the reference study, but were included in our models, since cell counts are recognised confounders in our data. Sensitivity analyses were, however, performed by including data on chronic medication use (as a proxy for disease) as well as excluding cell count adjustments. These analyses did not result in any discernible differences in findings (inclusion of medication use: regression slope = 13.0, *p* = 4.5 × 10^−30^, exclusion of cells: regression slope = 12.4, *p* = 1.7 × 10^−32^). There were no genome-wide significant or novel age-DNAm associations in our cohort (Additional file 2g).

## Discussion

Our primary analysis focussed on the replication of relevant EWAS literature in 120 Batswana men from the PURE-SA-NW cohort. Secondary analysis included the discovery of novel findings, either investigated for the first time on the EPIC array, or with the 450K probes incorporated in the EPIC array that had not previously been associated with these traits.

Overall, the 95% CI of effect estimates for 86% (4730 out of the 5498 CpG-trait association tests) of the PURE-SA-NW associations overlapped with previously reported findings, and a further 13% (720 out of the 5498 CpG-trait association tests) were directionally uniform. Generally, larger effect sizes were observed in the PURE-SA-NW data than those of the reference studies. Although the reason for differing effect sizes cannot be answered definitively, given the small sample size, the degree of association seems to be related to population-specific differences. Only ~ 1% of our findings (48 out of the 5498 CpG-trait association tests, including 44 unique CpGs) were directionally inconsistent with its compared association reported in the reference study. No data quality concerns were observed for any of these directionally contradicting findings. Of the 44 CpGs, 36 have mQTLs [24, 25] for which population differences in MAFs have been observed by the 1000 genomes project [26].

Overall, these results indicate general consistency in epigenome-wide associations among ethnicities, but ancestry may be important in up to 14% of the tested associations. This is supported by the fact that regardless of the similarity in traits measured among groups, the associations observed in PURE-SA-NW data consistently reflected those reported in AA better than in EU/EA cohorts and better in EU than IA in the case of methylation-BMI associations. Furthermore, eight novel associations between the methylation of 450K array probes, present on the EPIC platform, and alcohol consumption are reported in the Batswana South Africans that were not previously observed in populations of different ancestral origins. These population distinctions indicate the value of ethnic diversity in epigenetic research.

The only trait for which we were unable to replicate any associations was age. Apart from the reference study for age being the smallest of the reference studies included (*N* = 498), there were also clear differences in the pre-processing, data normalisation and EWAS approach followed between PURE-SA-NW and Florath et al. [19]. The reference cohort’s analyses were restricted to a pre-selected set of 200 CpGs, the methylation levels of which were normalised using Box–Cox transformations. A mixed regression model with plate and BeadChip as random effects was used. For the PURE-SA-NW data, however, we employed a functional normalisation strategy on the raw methylation data of all the EPIC BeadChip probes, followed by linear regression where surrogate variables were adjusted for as fixed effects to control for possible unaccounted variance. Our findings remained directionally consistent with the reference study’s, with the average difference in effect size amounting to 0.87% methylation change per year increase in age (calculated as the percentage difference between the average of the 152 tests’ absolute regression βs of the PURE-SA-NW vs. Florath et al [19] results).

In terms of findings related to the EPIC array, 11 genome-wide significant alcohol associations are reported here. An additional eight genome-wide significant alcohol associations were observed for 450K probes present on the EPIC array. Alcohol consumption contributed to a large portion of the variance in the methylation of these probes, as well as, when reversed, the probes to the variance in alcohol consumption. Previous 450K CpG-alcohol associations have been used successfully to identify risky and heavy drinkers [5]. Our sample size did not allow stratification of alcohol intake, but we expect the addition of the alcohol-associated EPIC probes reported here to enhance the discriminatory potential of the current methylation-based biomarker of alcohol consumption [5]. The variance explained by these findings and their usefulness as potential biomarkers warrant replication in large and ethnically diverse cohorts. Larger sample sizes and ethnic diversity will also permit further exploration of the biological basis of these findings and their potential application in NCD-related epigenetic research.

The strengths of this study are the expansion of current data, both by using the EPIC array and investigating a novel study population, after first being able to observe similar findings to those from independent, highly powered, previously replicated literature. The overall consistency between effect sizes is reassuring, not only in terms of the comparability of the PURE-SA-NW data with previous findings, but also the consistency in the effect size and explained variability of novel associations compared to previous EWASs on similar traits [5, 9, 20, 21]. The efficacy of the enhanced coverage of the EPIC array, to uncover new associations with a range of traits, is shown in our study, even with our limited sample size. We motivate the use of this array in future large-scale analyses, as it is likely to add to the variance that can be explained using methylation markers and also to identify novel sites that may be important in prediction, risk stratification or understanding causal disease pathways.

In this study, however, the corresponding limitation to doubling the coverage of the 450K array was the relative loss of statistical power, given our sample size. The lack of power resulted in wide regression CIs for most association estimates that limited our capacity for the fine scale inference of findings. We were able to comment on general patterns and large differences, but we do not know whether more subtle differences between population subgroups exist. Furthermore, the unavailability of genomic data in our cohort and the absence of data on Southern African populations in the 1000 genomes’ database restricted our ability to evaluate MAF differences between the reference and Batswana South African groups. We are, therefore, unable to quantify the relative contribution of genetic compared to environmental factors in the associations and association differences observed. The overall congruence in replication results between cohorts—even when large differences in phenotypes are demonstrated—does, however, suggest that these associations might be the result of genetic architecture rather than environmental differences, which we expect to affect the investigated traits as well.

The inclusion of only one sex also limits this study in that no assumptions can be made regarding the generalisability of these results to black South African women. However, because all the reference studies we replicated contained mixed-gender data, there are likely not major differences in these associations between the sexes.

## Conclusions

This study reports that up to 86% of the previously reported epigenome-wide associations observed in other ethinicities are present in this black male South African population. While acknowledging the value of ethnic-specific genomic data, our results support the notion that current blood-based 450K EWAS findings can largely be extrapolated to under-represented ethnicities for whom epigenetic data are not yet available. However, the population-specific differences in up to 14% of the CpGs tested, together with the unique associations reported here, do motivate the inclusion of a diversity of ethnic groups in epigenetic association studies. Investigating multi-ethnic data in epigenome-wide studies should be considered the golden standard.

## Methods

### Study design

This study was performed on a sub-sample of individuals participating in the international PURE study [27]. The PURE study includes sub-cohorts across the world, including one comprising individuals residing in the NW province of South Africa. This sub-cohort represents a single, self-reported ethnicity, Batswana South Africans, who were born and still reside in the NW province of South Africa. Detailed descriptions of the international and PURE-SA-NW cohorts have been published previously [27, 28].

PURE-SA-NW data were collected in 2005, 2010 and 2015. A total of 126 participants were randomly selected for the current investigation, from a group of 990 individuals who took part in the 2015 PURE-SA-NW data collection. Eligibility depended on the following inclusion criteria: availability of bio-samples, testing negative for the human immunodeficiency virus at the time of data collection and male sex. These criteria were incorporated to eliminate confounding by sex and CD4 cell counts in a study with already limited power. The participants included in this study are referred to as the PURE-SA-NW cohort in this manuscript.

### Data collection

Height and weight were quantified using a stadiometer and an electronic scale. BMI was calculated as weight per unit height squared (kg/m^2^). WC was measured at the appropriate landmarks, by qualified anthropometrists using a steel tape.

An adapted physical activity index questionnaire was used to gather data to calculate a physical activity index [29]. Alcohol intake (g/day) was determined from a quantitative food frequency questionnaire adapted and validated for use in this population [30]. Participants reported the amount, frequency and any relevant description of the alcoholic drinks they had consumed in the preceding month. Data were processed to an amount in g/day, based on the South African food composition tables using FoodFinder3^®^ software (available from http://foodfinder.mrc.ac.za). Smoking status (current, former or never) was self-reported, using a standardised questionnaire. When used as a covariate, smoking and drinking status were dichotomised into *never* and *ever* groups, with former smokers/drinkers included in the *ever* category. When investigated as the EWAS exposure, smoking status and alcohol consumption were classified according to the classification used in the reference studies.

Fasting blood samples were collected and handled as described previously [31]. High-sensitivity CRP and fasting blood lipids (TC, LDL-C, HDL-C, TG) were quantified using the Cobas^®^ Integra 400 (Roche^®^ Clinical System, Roche Diagnostics, Indianapolis, IN, USA).

### DNAm data generation and processing

Whole blood intended for the isolation of genomic DNA was collected in 9 mL Tempus tubes (Applied Biosystems™, Foster city, CA, USA) at the same time as blood used for the quantification of all other phenotypes. Tubes were vortexed for 10 s prior to storage in a − 20 °C freezer for up to 5 days, after which samples were transferred to cryostorage (− 80 °C) until analysis. DNA was isolated using QIAGEN Flexigene DNA extraction kits (QIAGEN^®^ Valencia, CA, USA). The manufacturer’s protocol was followed with minor modifications.

Upon extraction, the picoGreen^®^ dsDNA quantitation assay (Invitrogen™, Carlsbad, CA, USA) was used to quantify DNA. Five hundred nanograms DNA from each participant was bisulphite-converted using the Zymo EZ DNAm™ kit (Zymo Research, Irvine, CA, USA), followed by genome-wide DNAm profiling on the Illumina Infinium MethylationEPIC BeadChip according to the manufacturer’s protocol (Illumina^®^, San Diego, CA, USA).

Samples were randomised across slides to minimise the possibility of confounding by batch. Raw signal intensity data were processed from .idat files using functional normalisation as described by the R package *meffil* [32]. The quality threshold for samples and probes was set at 95%. All probes or samples with a detection *p* value > 0.01 for more than 5% of the evaluated measures were excluded. Six samples were removed on account of low quality: four samples because of a proportion of undetected probes above the quality control (QC) threshold and two with outlying control probes. Probes failing QC were removed prior to data normalisation (*N* = 8343). Eventually 857,516 probes and 120 individuals were included in subsequent data normalisation and analysis. Principal component analysis of the control probes identified 12 principal components to be included in the functional normalisation. In addition, *slide* was specified as a random effect to be included to address batch variance. Sample cell fractions (B cells, CD4 and CD8 T cells, neutrophils, monocytes and natural killer cells) were estimated using the IDOL optimised L-DMR library for whole blood samples [23].

### Identification of reference data using the EWAS catalogue

Data we sought to replicate were extracted from the EWAS catalogue (http://www.ewascatalog.org, date of access: 27 April 2019). The EWAS catalogue indexes EWAS studies performed in a study sample of at least 100 individuals for whom at least 100,000 CpGs were available genome-wide. Only associations with *p* < 1 × 10^−4^ are included in the catalogue.

Data from the catalogue were pruned according to the following criteria: (i) the EWAS catalogue trait had to be available in the PURE-SA-NW study cohort in a comparable unit; (ii) methylation-trait associations had to be replicated (below a *p* value threshold of 1 × 10^−4^) in at least one independent cohort, regardless of tissue, to reduce the possibility of including false positive findings from among the reference studies; (iii) the DNA had to have been extracted from a blood-based sample; (iv) DNAm had to be reported in Beta units; and (v) an effect estimate (β) and standard error had to be available for each association. Traits that fitted these criteria were age, alcohol consumption, smoking, BMI, WC, CRP, HDL-C, LDL-C, TG and TC. To simplify data analysis, we attempted to replicate results from the largest study indexed by the EWAS catalogue for each investigated trait only. The results reported in each replication sub-section make reference to the particular study used for comparison, which would have been the largest EWAS included in the catalogue at the time of writing.

### Statistical analysis

Statistical analysis was conducted using R 3.4.3 [33]. The normality of trait data was assessed using Shapiro-Wilks tests. Linear regression models were used to identify epigenome-wide associations using the *meffil* [32] and *ewaff* (https://github.com/perishky/ewaff) packages. DNAm was modelled as a β value between 0 and 1, representing the ratio of methylated to unmethylated probes. The relative contribution of exposures to the variance of outcome variables was determined using the *relaimpo* package’s *lmg* metric from the *calc.relimp* function applied to linear models.

For the replication analysis, because of the small sample size of the PURE-SA-NW study population and, therefore, limited power, replication of previously published results focusses on the size and direction of effect sizes rather than comparison of *p* values. Associations were considered replicable when the 95% CI of the regression β of the reference and the PURE-SA-NW cohort overlapped. Most reference studies extracted from the EWAS catalogue adjust regression models for ‘technical variation’. In PURE-SA-NW, surrogate variables were added to all models to reduce any unknown or unmeasured confounding [34]. The *sva* and *generate.confounders* functions within the *meffil* and *ewaff* packages estimated the surrogate variables that were included in each model based on the method described by Leek and Storey [34]. Annotation data were obtained from *meffil*.

For the investigation of novel findings, only associations with *p* < 9.4 × 10^−8^ were considered genome-wide significant [15]. Within our cohort, we estimated 80% power to detect a 5% difference in methylation at this threshold for 69% of the EPIC probes, assuming an alpha level of 0.05 and 530,639 independent tests [15]. Packages used in analyses, in addition to those already specified, include *BaseR*, *dplyr*, *FlowSorted.Blood.EPIC*, *ggplot*, *IlluminaHumanMethylationEPICanno.ilm10b2.hg19*, *minfi*, *readxl* and *xlsx.*

## Supplementary information

**Additional file 1.** EWAS test statistics (PURE-SA-NW vs reference study) for: (a) alcohol consumption; (b) smoking status; (c) BMI; (d) WC; (e) lipids; (f) CRP and (g) age.

**Additional file 2. **Epigenome-wide associations with *p* < 1 × 10^− 4^ in the PURE-SA-NW study for: (a) alcohol consumption; (b) smoking status; (c) BMI; (d) WC; (e) lipids; (f) CRP and (g) age.

## Data Availability

The data that support the findings of this study are available upon reasonable request and with the permission of the Health Research Ethics Committee of the North-West University and the principal investigator of the PURE-SA-NW study, Prof. I.M. Kruger (lanthe.kruger@nwu.ac.za) at the North-West University’s Africa Unit for Transdisciplinary Health Research.

## Abbreviations

AA: African American
BIOS: Biobank-based Integrative Omics Studies
BMI: Body mass index
Chr: Chromosome
CpGs: Cytosine-phosphate-guanine sites
CRP: C-reactive protein
DNAm: DNA methylation
EA: European American
EU: European
EWAS: Epigenome-wide association study
HDL-C: High-density lipoprotein cholesterol
IA: Indian Asian
IQR: Inter-quartile range
LDL-C: Low-density lipoprotein cholesterol
MAF: Minor allele frequency
mQTLs: Methylation quantitative trait loci
NCD: Non-communicable disease
PURE-SA-NW: South Africa, North-West arm of the Prospective Urban and Rural Epidemiology study
QC: Quality control
TC: Total cholesterol
TG: Triglycerides
UTR: Untranslated region
